# Green synthesized plant mediated zinc oxide nanoparticles as potential nano phytotherapeutic agents for reactive oxygen species driven anticancer activity in HepG2 liver cancer cells

**DOI:** 10.3389/fonc.2026.1832928

**Published:** 2026-10-29

**Authors:** Anupama Sharma, Lalit Prasad, Sandeep Kumar, Khalid Umar, Mohd Sajid Ali, Sadanand Pandey

**Affiliations:** 1 Department of Chemistry & Sustainability, School of Basic Sciences, Galgotias University, Greater Noida, India; 2 School of Pharmacy, Sharda University, Greater Noida, India; 3 School of Chemical Sciences, Universiti Sains Malaysia, Gelugor, Pulau Pinang, Malaysia; 4 Department of Chemistry, College of Science, King Saud University, Riyadh, Saudi Arabia; 5 Faculty of Applied Sciences and Biotechnology, Shoolini University, Solan, India; 6 Department of Medical Research, China Medical University Hospital, China Medical University, Taichung, Taiwan

**Keywords:** cancer nanotechnology, green synthesis, liver cancer, nanomedicine, nano-phytochemicals, phytotherapy, plant-derived anticancer agents, reactive oxygen species

## Abstract

Green nanotechnology has emerged as a promising approach for the sustainable synthesis of metal oxide nanoparticles with potential applications in cancer therapy. In this study, aqueous extracts of *Galinsoga parviflora* (GP), *Ageratum conyzoides* (AC), and *Argemone mexicana* (AM) were employed as green agents for the biosynthesis of zinc oxide nanoparticles (ZnO-NPs). Characterization confirmed the formation of phase-pure, crystalline ZnO-NPs with a hexagonal wurtzite structure and predominantly spherical morphology. The mean particle sizes were 12.33 ± 5.58 nm (GP-ZnO), 12.05 ± 4.51 nm (AM-ZnO), and 10.32 ± 1.98 nm (AC-ZnO), with the overall observed size range of 5–36 nm. Zeta potentials ranging from −8.2 to −8.6 mV indicated weak electrostatic stabilization and a tendency for some aggregation, as observed in FE-SEM images. FT-IR confirmed ZnO formation with residual surface-associated plant-derived constituents, while XRD exhibited characteristic reflections corresponding to the (100), (002), (101), (102), (103), (110), and (112) crystal planes of wurtzite ZnO. The biosynthesized ZnO-NPs demonstrated favorable biocompatibility, exhibiting minimal haemolysis and negligible cytotoxicity against normal THLE-2 liver cells. GP-ZnO, AM-ZnO, and AC-ZnO-NPs exhibited concentration-dependent cytotoxicity against HepG2 carcinoma cells, with IC_50_ values of 60.25 ± 3.2, 68.35 ± 3.4, and 74.15 ± 2.6 μg/mL, respectively. Treatment markedly enhanced intracellular ROS and Annexin V^+^ cells, with GP-ZnO and AM-ZnO-NPs inducing 59.71% and 57.40% Annexin V^+^ cells, respectively, against 50.05% for Doxorubicin (Dox). Despite lower ROS levels than Dox, their higher apoptotic fractions suggest ROS-independent mechanisms may contribute to cytotoxicity. However, lack of PI staining prevented differentiation between late apoptosis and necrosis. Overall, plant-mediated ZnO-NPs show promise as sustainable candidates for further *in vivo* investigation and translational development in liver cancer nanotherapy.

## 1 Introduction

Cancer continues to exist as one of the top causes of human morbidity and mortality throughout the globe, with liver cancer being one of the most aggressive and least responsive type (1). Conventional treatment approaches still encounter considerable challenges due to systemic toxicity, multidrug resistance, inappropriate tumor selectivity, and high recurrence rates (2), highlighting the urge to look for effective, safer, and sustainable therapies. In recent decades, nanotechnology-based carriers biosynthesized using plant-derived bioactives have been widely recognized as an innovative approach for targeting cancer, enhancing therapeutic precision and efficiency.

Nanomedicine incorporates nanoparticles to improve drug solubility, bioavailability, stability, biodistribution, and active targeting of malignant cells. Nowadays, metal oxide-based nanoparticles (NPs) have drawn much interest due to their distinctive physical and chemical characteristics, along with favorable surface reactivity and biological compatibility (3, 4). Metallic NPs with high purity have a wide range of applications in antimicrobial therapy, biosensing, drug delivery as well as cancer therapy (5, 6). Specifically, zinc oxide-derived NPs (ZnO-NPs) are of considerable interest since they display encouraging selective cytotoxicity towards malignant cells along with biosafety, cost-effectiveness and ease of fabrication (7). Various conventional chemical methods like sol-gel, hydrothermal, chemical vapor deposition and chemical precipitation have been employed to synthesize ZnO-NPs (8, 9). While these techniques offer fine control over the crystalline architecture and particle dimensions, they often involve the usage of hazardous chemicals, toxic solvents, high energy consumption or produce ecologically harmful byproducts. Faced with these challenges, scientific researchers have been progressively redirected towards sustainable, less harmful green alternatives (9, 10).

Green synthesis of NPs commonly utilizes biological sources as capping and stabilizing agents. The plant-mediated synthesis is particularly advantageous due to its simplicity, scalability, low cost, and avoidance of complex microbial culturing procedures (11). Secondary metabolites abundant in plant extracts such as alkaloids, flavonoids, phenolic chemicals, terpenoids, amino acids, polysaccharides, and vitamins, can dramatically reduce the metal ions and stabilize the resultant nanostructures (12, 13). These phytochemicals not only promote NPs formation but also prevent NPs agglomeration and enhance their colloidal stability. Medicinal plants including *Aloe vera* (14), *Ziziphus jujube* (15), *Cuminum cyminum* (16), *Calotropis gigantea* (17), *Mangifera indica* (18), *Azadirachta indica* (19), and *Abelmoschus esculentus* (20) have been effectively employed for eco-friendly synthesis of ZnO-NPs.

*Galinsoga parviflora* (GP), *Ageratum conyzoides* (AC), and *Argemone mexicana* (AM), members of the Asteraceae family, represent promising yet largely underexplored plant sources for green nanomedicine-based applications. These common weed plants are widely distributed across India and other tropical and sub-tropical regions, and are rich in bioactive compounds including alkaloids, flavonoids, polyphenols and terpenoids with known antibacterial, antioxidant, anti-inflammatory, and anticancer properties (21–23). GP has earlier been employed for the biosynthesis of iron-oxide based NPs displaying moderate antimicrobial, photocatalytic, and dye degradation activities (24–26). Similarly, AM has been utilized for the green synthesis of iron oxide, silver, gold, NiO, and ZnO NPs exhibiting antioxidant and antimicrobial properties (27–30). Notably, the anticancer activity of AM-mediated nanoparticles has been reported only for gold NPs against colon cancer cells (31). Likewise, AC has been extensively exploited for the green synthesis of iron, silver, Cu/Cu_2_O, NiO, and ZnO-NPs, exhibiting promising antimicrobial attributes and photocatalysis (24, 26, 32, 33). However, reports on AC ZnO-NPs are limited with most of the studies confined to their antimicrobial applications (34, 35), while a recent study demonstrated the growth-boosting potential of biogenic ZnO-NPs (36). According to our extensive analysis, this is the first comparative study to evaluate the anticancer efficacy of ZnO-NPs biosynthesized from GP, AC and AM weed-like plants against Hepatocellular carcinoma (HCC), and to investigate their mechanism of action via ROS-mediated apoptosis.

HCC, commonly modelled *in vitro* using HepG2 cells, is among the most prevalent and lethal malignancies worldwide (37). Green-synthesized, ROS-generating ZnO-NPs offer a promising avenue within sustainable nanomedicine approach for targeting this particular type of cancer. In this study, we report a simple, efficient, and environmentally sustainable synthesis of ZnO-NPs using aqueous extracts of *GP*, *AC*, and *AM* weeds. FT-IR, XRD, FE-SEM, TEM, and EDS were employed to characterize the synthesized NPs and determine their structural, morphological, and elemental properties. Additionally, their anticancer efficacy against HepG2 liver cancer cells was subsequently utilizing MTT cytotoxicity tests, ROS detection and apoptotic assays.

## 2 Materials and methods

### 2.1 Materials

Zinc nitrate hexahydrate [Zn(NO_3_)_2_·6H_2_O; Sigma-Aldrich, USA] was used as the zinc precursor for nanoparticle synthesis. The reagents employed for biological evaluations included 3-(4,5-dimethylthiazol-2-yl)-2,5-diphenyltetrazolium bromide (MTT) for cytotoxicity assessment and 2,7-dichlorodihydrofluorescein diacetate (DCFH-DA) for the detection of intracellular reactive oxygen species (ROS), both procured from Sigma-Aldrich (USA). Annexin V-FITC Apoptosis and ROS Assay Kits were obtained from Thermo Fisher Scientific (USA) and Real-Gene Labs (India), respectively. The human hepatocellular carcinoma cell line HepG2 (HB-8065) and the THLE-2 cell line (CRL-2706), a transformed human hepatocyte cell line used as a control, were purchased from the American Type Culture Collection (ATCC, Manassas, Virginia, USA). For cell culture experiments, Dulbecco’s Modified Eagle Medium (DMEM), supplemented with 10% fetal bovine serum (FBS; South American origin; FB-1001/100) and 1% penicillin–streptomycin, was obtained from Biosera (Nuaille, France). All cell lines were routinely screened for mycoplasma contamination before the commencement of the experiments. All chemicals and reagents used in the study were of analytical grade and were used as received without further purification.

### 2.2 Preparation of plant leaf extracts

*G. parvifloras*, *A. conyzoides*, and *A. mexicanas* were collected from Hathras, U.P., India as well as Greater Noida campus of Galgotias University, U.P., India. The collected plant materials were identified at the School of Agriculture, Galgotias University, India, based on comparison of their vegetative and floral morphological characteristics with standard taxonomic descriptions in the Flora of India as previously reported (38, 39). Voucher specimens were submitted in the herbarium of the Galgotias University (specimen number GU/2025/021). Freshly harvested leaves were thoroughly washed under running water to get rid of all dust and surface particles, followed by cleaning in deionized (DI) water to wash out the residual impurities. To prevent the breakdown of thermolabile phytoconstituents, the leaves were shade-dried, then chopped and pounded into a coarse powder using a sterile mortar and pestle. For each specimen, 10 g of powdered leaf specimen was transferred into a 500 mL beaker along with the requisite volume of DI water, and the mixture was boiled for 10 min to extract bioactive phytoconstituents. After cooling to room temperature, the decoction was centrifuged at 5000 rpm for 15 min. This centrifugation step was repeated three times to obtain a clear, particle-free supernatant. The resulting extract was thoroughly decanted and stored at 4 °C (277.15 K) until further use.

### 2.3 Green synthesis of zinc oxide nanoparticles

For each plant-derived extract, 100 mL of freshly prepared leaf extract was mixed with 6.0 g of zinc nitrate hexahydrate [Zn(NO_3_)_2_·6H_2_O] under continuous stirring at 70 °C. Subsequently, 10 mL of 0.5 M sodium hydroxide (NaOH) solution was added dropwise with constant stirring. The pH of the reaction mixture was monitored continuously using a calibrated digital pH meter with an accuracy of ±0.01 and adjusted to 12.0 ± 0.1. The resulting mixture was maintained under continuous stirring for 1 h, during which the formation of a colloidal suspension indicated the nucleation and growth of the nanoparticles. The reaction mixture was then evaporated to remove the solvent, and the resulting solid residue was dried and gently pulverized into a fine powder using a mortar and pestle. Finally, the dried powder was calcined in a muffle furnace at 400 °C for 2 h to facilitate the formation of crystalline ZnO nanoparticles (ZnO-NPs). To ensure that the differences in the properties of nanostructures arose solely from variations in plant phytochemistry, identical synthesis conditions were applied to all three extracts. These parameters (100 mL extract, 6.0 g Zn(NO_3_)_2_·6H_2_O, 10 mL of 0.5 M NaOH, pH adjusted to 12.0 ± 0.1, 70 °C, 1 h of stirring, and calcination at 400 °C for 2 h) were established through preliminary pilot optimization studies and are consistent with established methodologies for plant-mediated ZnO-NPs (40). The temperature and stirring duration were chosen to promote controlled, homogeneous nucleation and adequate interaction between plant-derived capping biomolecules and the growing NPs, and calcination conditions ensured complete conversion of the precursor to crystalline ZnO without excessive NPs growth.

### 2.4 Characterization of ZnO-NPs

The morphological features and particle size were evaluated using Transmission Electron Microscopy (TEM) and Field Emission Scanning Electron Microscopy (FE-SEM). TEM analysis was performed using a Tecnai™ G2 20 (Thermo Fisher, USA) instrument, while FE-SEM and Energy Dispersive X-ray Spectroscopy (EDS) analyses were conducted using JEOL JSM-7610F Plus Schottky FE-SEM (JEOL Ltd., Peabody, MA, USA) and Zeiss EVO 50 equipped with SmartEDX (Zeiss, Mumbai, India). For imaging, 20 mg/mL of samples in acetone were sonicated for 20 min to obtain a homogeneous dispersion. A drop of the suspension was then spotted onto carbon tape (for FE-SEM and EDS) and onto a copper grid (for TEM) using the drop-casting method for analysis. The substrates were then dried at ambient temperature within 30 min and then imaged. Particle size measurements and size-distribution histograms were generated from TEM micrographs using ImageJ software (National Institutes of Health, USA), with the scale calibrated against the embedded scale bar of each image prior to measurement. Zeta potential analysis was performed using an SurPASS 3 Standard Electrokinetic Analyzer (Anton Paar, Austria, GmBH) to determine the surface charge of each NPs dispersed in DI water. FT-IR spectroscopy was employed to characterize the functional groups using a Thermo Scientific Fourier-transform infrared (FTIR) spectroscopy was performed using a Nicolet iS50 spectrometer equipped with an iD5-ATR accessory (Thermo Fisher Scientific India Pvt. Ltd., Mumbai, India). Spectral measurements were acquired over the wavenumber range of 4000–400 cm⁻¹ at a spectral resolution of 4 cm⁻¹. The crystalline structure of the synthesized nanoparticles was investigated by powder X-ray diffraction (XRD) using an X’Pert PRO diffractometer (Malvern Panalytical, Malvern, WR14 1XZ, UK). Approximately 25 mg of each powdered sample was uniformly mounted on a clean glass slide, and the diffraction patterns were recorded over a 2θ range of 30–70° with a total scanning time of 2 h. The resulting diffraction peaks were indexed and compared with standard reference data from the Joint Committee on Powder Diffraction Standards (JCPDS) database to confirm the crystal structure and assess the phase purity of the synthesized nanoparticles. The average crystallite size was calculated from peak FWHM using the Debye-Scherrer equation as reported earlier (41).


$$D=(\frac{\text{K}.}{\text{d}.\text{cos}})$$

where, D is crystallite size, k is proportionality constant, λ is X-ray wavelength of Cu-K_α_ radiation (1.54178 Å), θ is full width at half maximum (FWHM) of XRD peaks or Bragg’s angle.

### 2.5 ZnO-NPs-induced red blood cells hemolysis

The hemolytic potential of the synthesized ZnO-NPs was evaluated using an erythrocyte hemolysis assay adapted from the principles of ASTM F756-17(2025). The test was performed under static conditions using freshly collected anticoagulated blood. Briefly, the blood was diluted with calcium- and magnesium-free phosphate-buffered saline (PBS) to obtain the required test suspension. Briefly, 1 mL of ZnO-NP suspensions at concentrations of 6.25, 12.5, 25, 50 and 100 µg/mL was incubated with a standardized red blood cell (RBC) suspension (~2 × 10^8^ cells/mL) at 37 °C for 24 h, maintaining a final assay volume of 2 mL. Following incubation, the samples were centrifuged at 1300 × g, and the supernatants were carefully collected. The amount of haemoglobin released into the supernatant was quantified by measuring the absorbance at 540 nm. A 0.1% Triton X-100 solution was used as the positive control and was considered to represent 100% hemolysis, whereas untreated RBCs were used as the negative control. The percentage of RBC hemolysis was calculated using the specified equation and expressed relative to the 100% hemolysis control.


$$\text{Corrected}\;\%\text{ RBC lysis}=(\frac{{\text{Abs}}_{\text{S}}-{\text{Abs}}_{\text{c}}}{{\text{Abs}}_{T}-{\text{Abs}}_{\text{C}}})\times 100$$

Where, Abs_S_ = absorbance from cells incubated with the nano-formulation samples,

Abs_C_ = absorbance from the control (PBS),

Abs_T_ = absorbance in the presence of 0.1% Triton X-100.

### 2.6 *In vitro* cytotoxicity assessment by MTT assay

The cytotoxic potential of GP-ZnO, AM-ZnO, and AC-ZnO formulations against HepG2 cells was determined employing the 3,(4,5-dimethylthiazol-2-yl)-2,5-diphenyltetrazolium bromide (MTT) test (42). The HepG2 cells (1 × 10^4^ cells/well) were placed in 96-well culture plates for 4 hrs. Next, the cells were exposed to different concentrations of ZnO-NPs (0-150 µg/mL) for 24 hrs. Untreated cells were taken as the negative control group, while Doxorubicin (Dox) treated cells were taken as a positive control for reference. Afterwards, 20 µL of MTT solution (5 mg/mL in PBS) was pipetted into each well, and the mixture was then incubated for 3–4 hrs at 37 °C in a BOD incubator. After the media was aspirated, 100 μL of DMSO was added to bring the insoluble purple formazan crystals into solution. After briefly spinning the plates, the absorbance was quantified at 590 nm with the help of a microplate reader (Agilent BioTek Synergy neo2 Hybrid Multi-Mode Reader, Santa Clara, CA, USA) and cell viability was computed as per the equation below:


$$\text{Cell viability }(\%)=\;(\frac{\text{Mean absorbance of treated cells}}{\text{Mean absorbance of control cells}})\;\times \;100$$

where, mean optical density (OD) values of wells exposed to NPs represent the mean absorbance of treated cells. The mean absorbance of control cells represents the average OD values from untreated cells, serving as the baseline reference for 100% viability.

To evaluate the biosafety and cytotoxic potential of ZnO-NPs toward normal adult liver epithelial cells, similar experiments were conducted using the THLE-2 cell line. Dox-treated cells and untreated cells were used as the positive and negative controls, respectively. All experiments were performed in biological triplicates, and the results are presented as the mean ± standard deviation (SD). Dose–response data were analyzed using appropriate nonlinear regression models to determine the half-maximal inhibitory concentration (IC_50_) values.

### 2.7 Measurement of intracellular reactive oxygen species

Qualitative analysis of intracellular ROS production was assessed with DCFH-DA fluorescent assay. In brief, HepG2 cells were seeded in 6-well culture plates at a concentration of 1 × 10^5^ cells per well. After 4 hrs of adherence, the positive control group was treated with 100 µL of 0.080 mM hydrogen peroxide (H_2_O_2_) for 4 hrs to induce oxidative stress, while experimental groups were treated with ZnO-NPs (GP-ZnO, AC-ZnO, or AM-ZnO) at 50 µg/mL for 4 hrs. Following the treatment, cells were gently washed with PBS and subsequently incubated with 8 µM DCFH-DA in serum-free medium for 30 min at 37 °C in the dark to prevent photo-oxidation of the fluorescent probe. Detection of fluorescence was realized with the excitation wavelength of 488 nm and emission wavelength of 530 nm (43). Similar experiment was performed for the quantitative analysis of intracellular ROS levels using the Real-Gene ROS Assay Kit (Real-Gene Labs, Ghaziabad, India), as per the manufacturer’s directives. The only modification was the use of multiple concentrations of ZnO-NPs bracketing the IC_50_ value to evaluate dose-dependent ROS generation. Fluorescence intensity was appraised employing a microplate ELISA reader (Agilent BioTek Synergy, USA) at excitation and emission wavelengths of 488 and 530 nm, respectively. Optical density (OD) measurements were utilized to adjust the fluorescence readings to cell density after background fluorescence was removed using blanks. The data were expressed as relative fluorescence units (RFU), and computed as mean ± SD.

### 2.8 Annexin V-FITC apoptosis assay

Apoptosis in HepG2 cells following exposure to the synthesized NPs was assessed using the Annexin V-FITC Apoptosis Detection Kit (Thermo Fisher Scientific, Carlsbad, CA, USA) according to the manufacturer’s instructions. Briefly, HepG2 cells were seeded in 6-well plates at a density of 3 × 10^5^ cells/well and incubated overnight at 37 °C in a humidified atmosphere containing 5% CO_2_ to allow cell attachment. The cells were subsequently exposed for 24 h to GP-ZnO, AM-ZnO, and AC-ZnO at their respective IC_50_ concentrations. Dox-treated cells were included as a positive treatment control, whereas untreated cells served as the negative control. After treatment, both the conditioned culture media and harvested cells were collected and pooled, followed by centrifugation at 300 × g for 5 min at 4 °C. The resulting cell pellets were washed twice with ice-cold 1× phosphate-buffered saline (PBS) and resuspended in 1× Annexin V binding buffer at a concentration of approximately 1 × 10^6^ cells/mL. Subsequently, 100 µL aliquots of the cell suspension were incubated with 5 µL of Annexin V-FITC conjugate for 15 min at room temperature in the dark. Following staining, 400 µL of Annexin V binding buffer was added to each sample. Flow-cytometric analysis was performed within 1 h of staining using a BD FACSLyric flow cytometer (BD Biosciences, San Jose, California, USA).

Fluorescence was detected through the FITC channel (530/30 nm), and the instrument voltage/gain settings were optimized using unstained controls. A total of 50,000 events were acquired for each sample. Apoptotic responses were quantified and reported as the percentage of Annexin V-positive (Annexin V^+^) cells and median fluorescence intensity (MFI). All acquired events were initially visualized to assess the overall cell population. Intact cells were first gated using FSC-A vs SSC-A to exclude debris, non-cellular events, and dead cells. Doublets and cell aggregates were subsequently excluded using FSC-A vs FSC-H to ensure analysis of single cells only. Apoptotic cells were identified based on phosphatidylserine (PS) externalization detected by Annexin V-FITC fluorescence, with unstained cells used to define the negative fluorescence region. The percentage of Annexin V^+^ cells was calculated within the singlet population for each treatment group.

### 2.9 Statistical analysis

All experiments were conducted independently using three biological replicates, and the results are presented as mean ± standard deviation (SD). Statistical analyses were carried out using GraphPad Prism software (version 10.0; GraphPad Software, San Diego, CA, USA). Dose–response data were analyzed using nonlinear regression of concentration–response curves to determine the half-maximal inhibitory concentration (IC_50_) values. Differences among multiple treatment groups were evaluated using one-way analysis of variance (ANOVA), followed by Tukey’s honestly significant difference (HSD) *post-hoc* test for multiple pairwise comparisons. Adjusted p-values were used to account for multiple comparisons, with p < 0.05 considered indicative of statistical significance.

## 3 Results and discussion

### 3.1 Plant-mediated green synthesis of ZnO-NPs

The aqueous extracts of *A. mexicana, A. conyzoides*, and *G. parviflora*, respectively, were used in an ecologically friendly, phytochemical-mediated precipitation approach to effectively produce AM-ZnO, AC-ZnO, and GP-ZnO NPs. The formation of ZnO-NPs was clearly indicated by the emergence of a light yellow or off-white precipitate throughout the reaction process (**Figure 1**). This was followed by calcination to produce a fine crystalline powder. Phenolic chemicals, flavonoid compounds, terpenoid compounds, alkaloid compounds, sterols, β-carotene, α-tocopherol, and fatty acids present in the plant extracts have been proposed to play dual roles in the NPs formation. These bioactive compounds are naturally occurring reducing agents that reduce zinc precursor ions (Zn^2+^) to intermediate, Zn(OH)_2_, which is subsequently converted to crystalline ZnO upon calcination. Also, these phytoconstituents act as stabilizing and capping agents that inhibit uncontrolled formation and aggregation of NPs (44). Coordination of Zn^2+^ ions with -OH, -C=O, and -NH_2_ functional moieties of plant metabolites is proposed to promote controlled nucleation and limit particle growth by adsorbing onto the NPs surface, with polyphenols and other antioxidant compounds likely assisting in this process. Zinc retains its (+2) oxidation state throughout, and no electron-transfer reduction of Zn^2+^ occurs, distinguishing this mechanism from noble-metal nanoparticle syntheses (e.g., Ag^+^ → Ag^0^), where phytochemicals act as genuine reducing agents. Stabilization of NPs by adsorbed organic compounds may also occur via steric hindrance, which can help limit aggregation and support more uniform particle size distribution (45).

**Figure 1:**
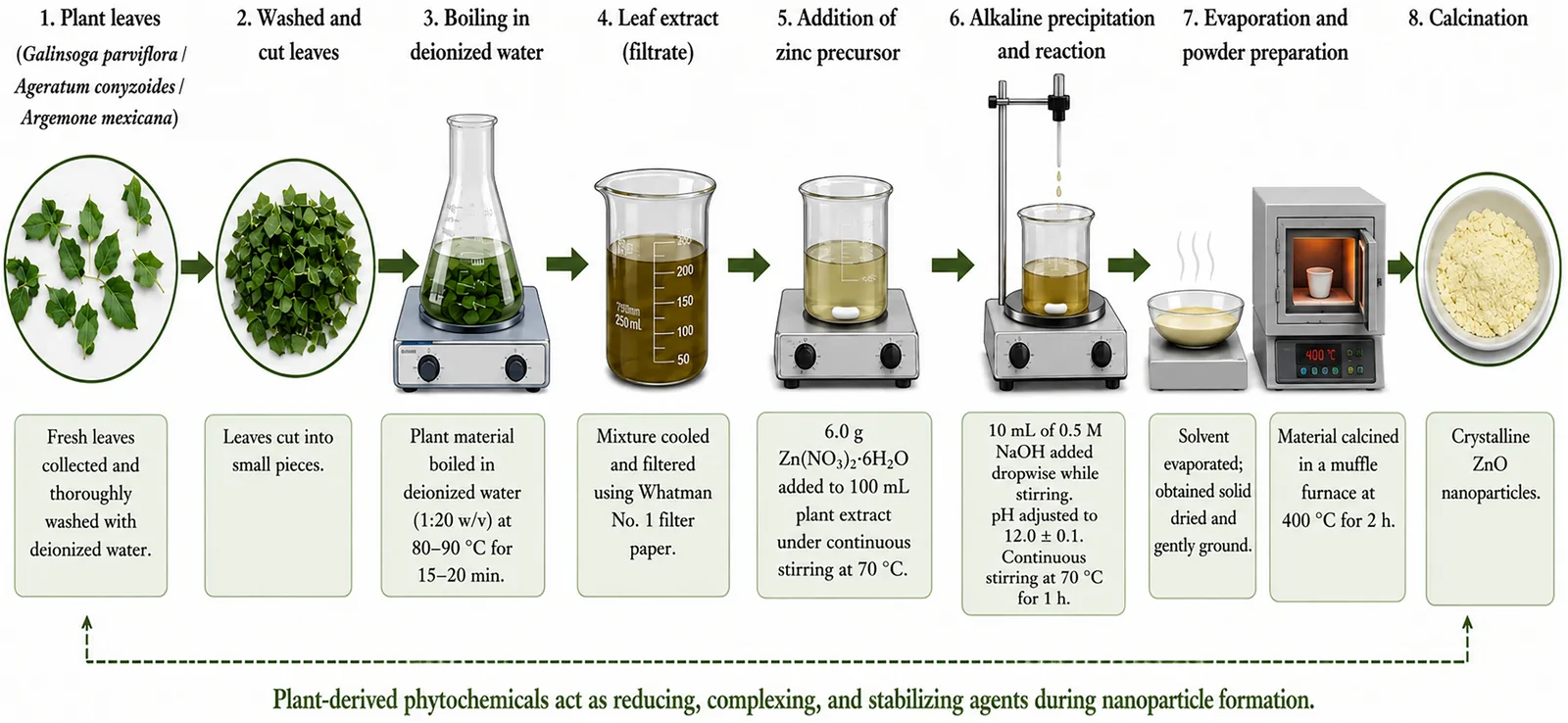
Schematic representation of the phytochemical-mediated green synthesis of ZnO nanoparticles, illustrating plant extract preparation, the addition of zinc nitrate hexahydrate [Zn(NO_3_)_2_·6H_2_O] as the zinc precursor, NaOH-mediated alkaline precipitation and pH adjustment, drying, and calcination to obtain crystalline ZnO-NPs. Plant-derived phytochemicals are proposed to act as reducing, complexing, and stabilizing agents during nanoparticle formation.

### 3.2 Fourier transform infrared analysis

The FTIR spectra (**Figure 2a**) of AM, AC, and GP extracts reveal common absorption bands, indicating the presence of polyphenolic and other bioactives. A broad band in the range of 3200–3500 cm⁻¹ confirms hydrogen-bonded hydroxyl (-OH) groups associated with phenolics and flavonoids in all extracts (46). However, variations in peak intensity and distribution within the fingerprint region (1500–500 cm⁻¹) highlight compositional differences among the plants. AM shows major characteristic peaks at 3428, 2928, and 1598 cm⁻¹, indicating stretching vibration of the -OH moiety, C-H stretching vibration of alkanes and C=C stretching vibrations of aromatic rings, respectively (30, 47, 48). In AC extract spectra, peaks at 3270 and 2920 cm⁻¹ may correspond to O-H stretching of phenols, alcohols and carboxylic acids, while the 1616 cm⁻¹ band can be attributed to C=O stretching of amides, carboxylic acids, or flavonoids (49, 50). Further, an FT-IR peak at 1262 cm⁻¹ could be identified as C-O stretching of aryl ether (50) and a distinct band at 1045 cm⁻¹ may be due to alcohols, ethers, or polysaccharides, suggesting the presence of flavonoids, tannins, and ether-linked compounds (49). In contrast, GP extract displays peaks at 3356, 1400, and 1094–1004 cm⁻¹, may reflect O-H stretching of phenolic groups, C = C aromatic stretching, and C-O-C vibrational frequencies of glycosidic linkage, respectively (26). Altogether, the spectra confirm that all extracts are rich in antioxidant phytochemicals.

**Figure 2:**
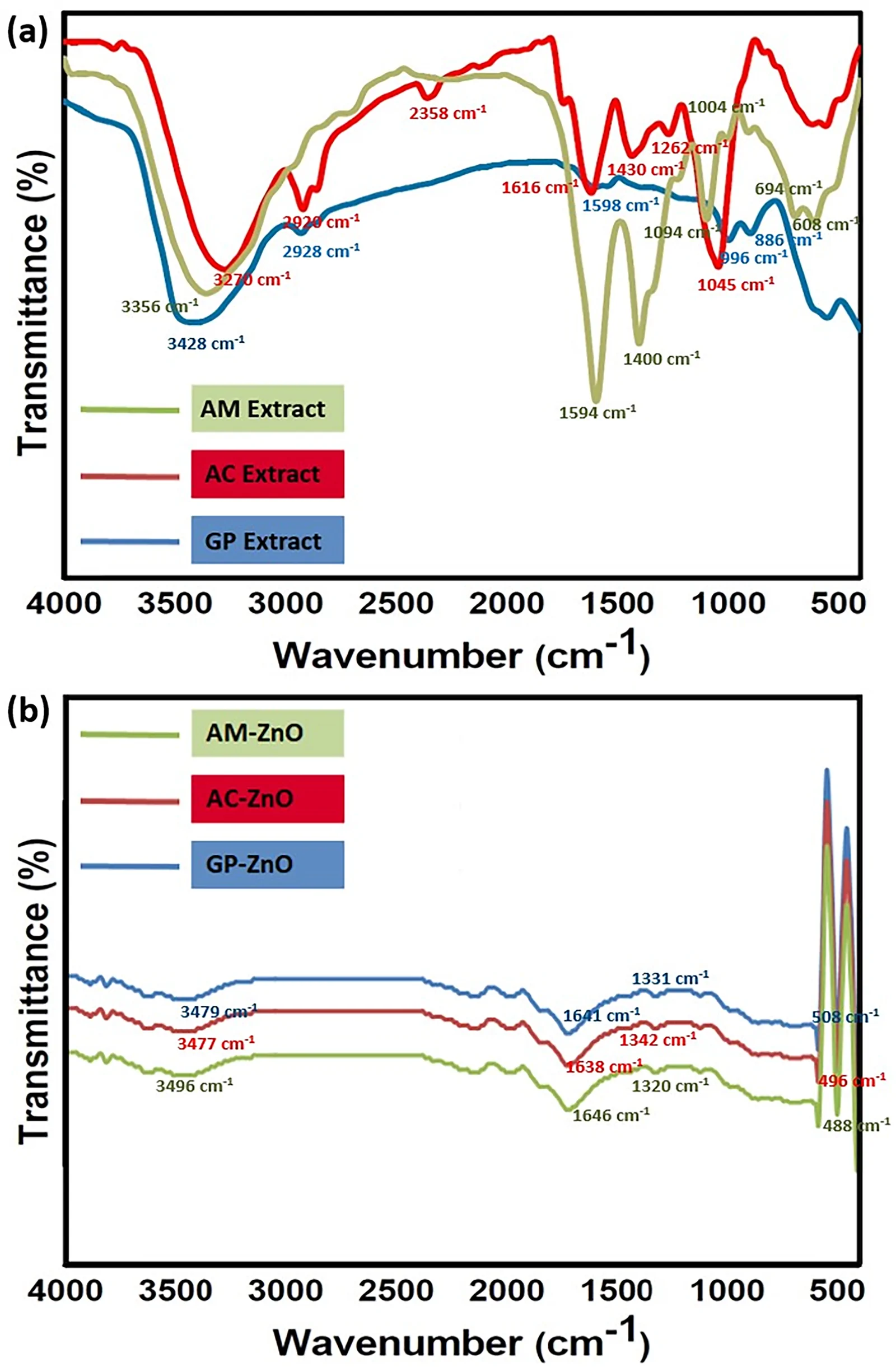
**(a)** FT-IR spectra of *Argemone mexicana*, *Ageratum conyzoides*, and *Galinsoga parviflora* extracts. **(b)** FT-IR spectra of biosynthesized GP-ZnO, AM-ZnO, and AC-ZnO NPs, showing the major annotated absorption bands associated with surface hydroxyl groups, H-O-H bending, residual nitrate species, and Zn-O vibrational frequencies.

For ZnO-NPs, a broad, strong absorption band corresponding to O-H stretching vibrations (**Figure 2B**) was observed at 3479 cm⁻¹ (GP-ZnO), 3477 cm⁻¹ (AC-ZnO), and 3496 cm⁻¹ (AM-ZnO) (51). Such bands are typically associated with surface -OH groups is suggestive of physisorbed atmospheric moisture and extract impurities (52). A minor contribution from residual -OH-bearing phytochemicals cannot be excluded but is not confirmed as a major contributor on the basis of this band alone (53). The absorption peaks at 1641 cm⁻¹ (GP-ZnO), 1638 cm⁻¹ (AC-ZnO), and 1646 cm⁻¹ (AM-ZnO) may be assigned to H-O-H bending vibrations (54). Similarly, bands at 1331 cm⁻¹ (GP-ZnO), 1342 cm⁻¹ (AC-ZnO), and 1320 cm⁻¹ (AM-ZnO), which could be interpreted as C-N vibrations of nitrate (NO_3_^−^) ions, and are more plausibly attributed to residual surface-adsorbed nitrate species (asymmetric NO_3_^−^ stretch), consistent with the use of zinc nitrate hexahydrate as precursor (55). A sharp and well-defined absorption band in the low-wavenumber region (450–600 cm⁻¹), attributed to Zn-O stretching vibrations, confirms the successful formation of ZnO-NPs. The presence of Zn-O vibrational bands is a clear indication of successful conversion of zinc precursors into crystalline ZnO (41). A weak similarity between ZnO and extract spectra is observed through residual O-H bands, primarily due to surface hydroxyl groups and adsorbed moisture, with only minor phytochemical residues remaining after calcination.

### 3.3 Energy dispersive x-ray spectroscopy analysis

EDS analysis provides critical information about the elemental profile and homogeneity of the biosynthesized NPs, which is vital in their biomedical applications, since impurities impacts cytotoxicity and biocompatibility. EDS spectra (**Figures 3a–c**) showed clear, well-resolved peaks comparable to zinc (Zn) and oxygen (O) in all three formulations, alongside a residual carbon (C) signal, most likely arising from adventitious surface carbon and/or incompletely combusted organic residues from the plant extract despite calcination. The measured atomic percentages and associated instrument-reported measurement uncertainty (1σ) are summarized in **Table 1**. ZnO atomic ratios were found to be 46.35:20.02 for GP-ZnO, 58.01:32.41 for AM-ZnO, and 45.78:24.40 for AC-ZnO NPs.

**Figure 3:**
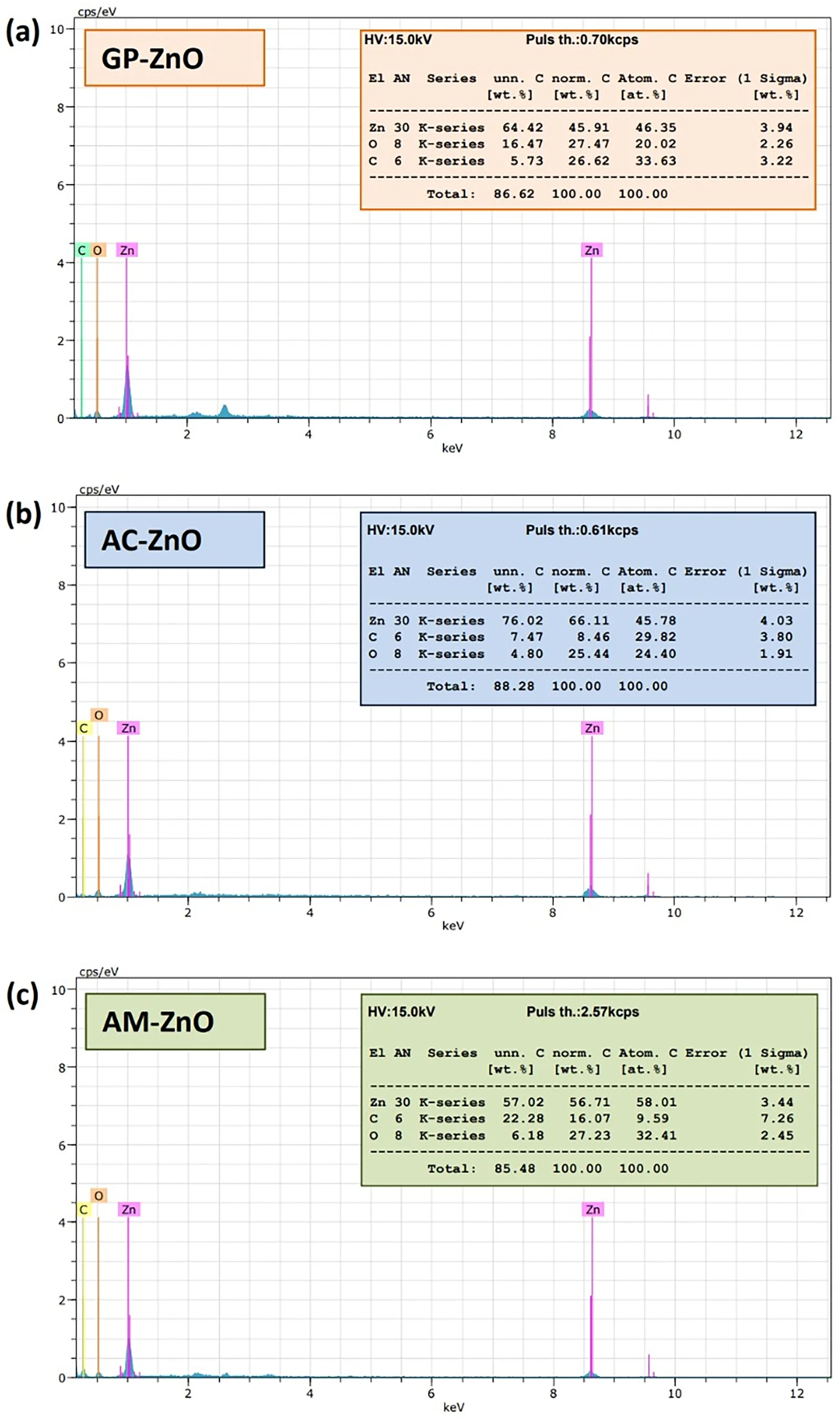
Elemental analysis (Zn and O) of all biosynthesized NPs; *GP-*ZnO **(a)***, AM-*ZnO **(b)**, and *AC-*ZnO NPs **(c)**.

**Table 1:** EDX-derived elemental composition and Zn:O stoichiometric ratio of GP-ZnO, AC-ZnO, and AM-ZnO nanoparticles, with instrument-reported measurement uncertainty (1σ).

Sample	Zn (Atomic %)	O (Atomic %)	C (Atomic %)	Zn error (wt%, 1σ)	O error (wt%, 1σ)	C error (wt%, 1σ)	Zn:O atomic ratio
GP-ZnO	46.35	20.02	33.63	3.94	2.26	3.22	2.32:1
AC-ZnO	45.78	24.40	29.82	4.03	1.91	3.80	1.88:1
AM-ZnO	58.01	32.41	9.59	3.44	2.45	7.26	1.79:1

Atomic % denotes atomic percentage obtained from energy-dispersive X-ray spectroscopy (EDS). The reported analytical errors represent one standard deviation (± 1σ) of the elemental weight percentage measurements.

The stability and structural integrity of the produced NPs are further supported by the elements zinc and oxygen as well as the lack of undesirable metallic impurities. These values are in line with previously published studies on plant-mediated ZnO-NPs synthesis (15, 56, 57). The Zn:O ratios deviate from the ideal 1:1 stoichiometry of bulk ZnO, consistent with well-recognized limitations of EDX for light-element quantification, including systematic oxygen under-detection, spectral overlap between C and O peaks, and higher inherent measurement uncertainty for light elements relative to Zn (58, 59). Even with this discrepancy, the predominance of Zn and O as the two main elemental peaks, alongside the lack of unexpected metallic contaminant peaks, signifies effective transformation of the Zn precursor to ZnO coupled with a comparable phase observed via XRD as wurtzite crystalline structure (**Figure 4**) and Zn-O vibration confirmed using FT-IR spectroscopy (**Figure 2**).

**Figure 4:**
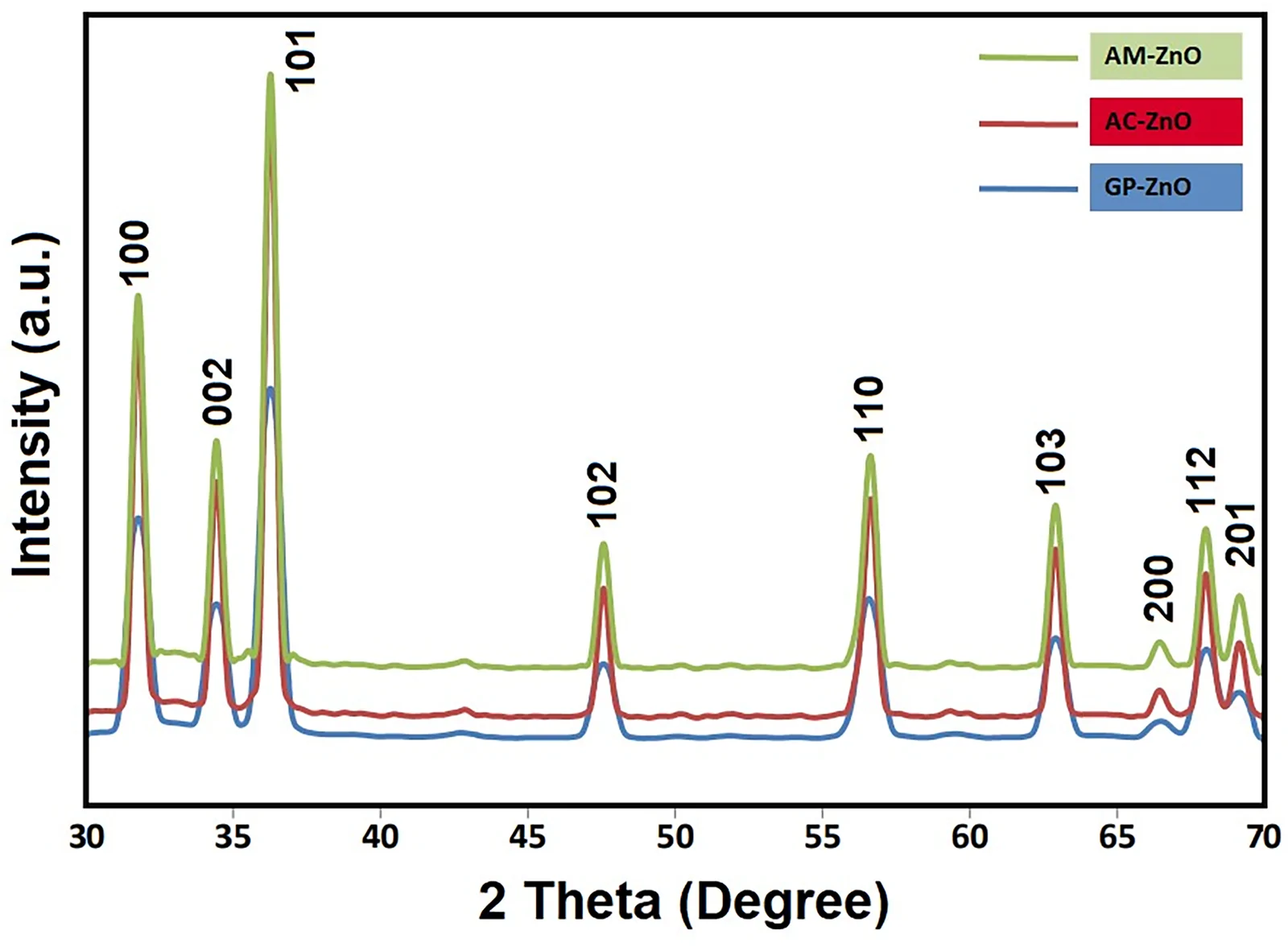
X-ray diffraction (XRD) patterns of biosynthesized GP-ZnO, AC-ZnO, and AM-ZnO NPs showing characteristic diffraction peaks indexed to the (100), (002), (101), (102), (110), (103), (200), (112), and (201) planes of the hexagonal wurtzite ZnO structures (JCPDS card no. 36-1451).

### 3.4 Powder x-ray diffraction analysis

Powder XRD analysis was used to study the crystal lattice organization and phase purity of the biosynthesized GP-ZnO, AC-ZnO, and AM-ZnO NPs. Diffraction patterns (**Figure 4**) were recorded and indexed against the standard hexagonal wurtzite ZnO reference pattern (JCPDS card no. 36-1451). All three formulations exhibited diffraction peaks indexed to the (100), (002), (101), (102), (110), (103), (200), (112), and (201) planes of hexagonal wurtzite ZnO, in close agreement with the JCPDS reference pattern (**Table 2**).

**Table 2:** Indexed crystallographic planes (hkl) and their corresponding 2θ values showing theoretical (JCPDS Reference 36-1451) and experimental diffraction angles for GP-ZnO, AC-ZnO, and AM-ZnO NPs, confirming phase purity and hexagonal Wurtzite crystal structure obtained through green synthesis.

hkl planes			2θ degrees		
	JCPDS reference 2θ (°)	d-spacing (Å)	Observed values		
			GP-ZnO	AC-ZnO	AM-ZnO
(100)	31.77	2.814	31.7	31.7	31.7
(002)	34.42	2.603	34.4	34.4	34.4
(101)*	36.25	2.475	36.2	36.2	36.2
(102)	47.56	1.913	47.5	47.5	47.5
(110)	56.67	1.624	56.6	56.6	56.6
(103)	62.93	1.478	62.9	62.9	62.9
(200)	66.37	1.407	66.4	66.4	66.4
(112)	67.97	1.377	67.9	67.9	67.9
(201)	69.10	1.358	69.1	69.1	69.1
FWHM, β (°)	–	–	0.45062	0.43010	0.74554
Crystallite size D (nm)	–	–	18.55	19.43	11.20

*(101) is the most intense peak and was used for Scherrer crystallite size calculation.

Additionally, the absence of additional diffraction peaks confirms successful conversion of the zinc precursor to phase-pure ZnO, with no evidence of secondary phases or unreacted precursor material. Similar peaks have been observed by other researchers (60, 61). The effective creation of phase-pure ZnO-NPs using the green synthesis method is confirmed by concordance between these diffraction patterns and the standard JCPDS reference data for hexagonal Wurtzite ZnO (62). The average crystallite size, calculated from the FWHM of the highest peak (101) using the Debye-Scherrer equation was 18.55 nm (GP-ZnO), 19.43 nm (AC-ZnO), and 11.20 nm (AM-ZnO) (**Table 2**). All samples exhibited sharp, well-resolved diffraction peaks, indicative of a well-ordered crystalline phase, which is relevant to nanomedicine applications given its influence on surface reactivity and the capacity of ZnO-NPs to generate reactive oxygen species (ROS). This confirmation of the hexagonal wurtzite structure agrees with the already reported semiconductor nature and oxidative stress inducing potential of ZnO in biological systems (63). Overall, the XRD analyses confirm the successful synthesis of high crystal phase pure ZnO-NPs with hexagonal Wurtzite structure.

### 3.5 Morphological analysis, size and zeta potential of ZnO NPs

ZnO-NPs morphology was examined using HR-TEM and FE-SEM in order to provide details about their morphology, structural uniformity, and nano-dimensions. The three synthesized nanoformulations exhibited predominantly spherical to quasi-spherical morphology, with varied degrees of aggregation, as shown in **Figure 5i**. Quantitative particle size analysis was performed by measuring individual particle diameters from the HR-TEM micrographs using ImageJ software (NIH), calibrated against the embedded scale bar for each image. GP-ZnO (**Figure 5ii**) showed a right-skewed distribution with a mode in the 8-10 nm range and a mean particle size of 12.33 ± 5.58 nm (n = 100), including a minority population of larger particles up to 36.04 nm. AC-ZnO showed a comparatively narrower, more symmetric distribution ca 9-11 nm, with a mean particle size of 10.32 ± 1.98 nm (n = 100). AM-ZnO showed a right-skewed distribution with a mode in the 5-7 nm range and a mean particle size of 12.05 ± 4.51 nm (n = 96). The comparatively broader distributions observed for GP-ZnO and AM-ZnO, relative to AC-ZnO, are consistent with the greater degree of particle aggregation observed in the corresponding TEM micrographs (**Figure 5iii**). Additionally, AM-ZnO shows close agreement between XRD and TEM sizing (ratio ≈ 1.08), consistent with a predominantly single-crystalline particle population, while GP-ZnO and AC-ZnO show a modestly smaller TEM mean relative to XRD, attributed to the difference between intensity-weighted and number-weighted size averaging.

**Figure 5:**
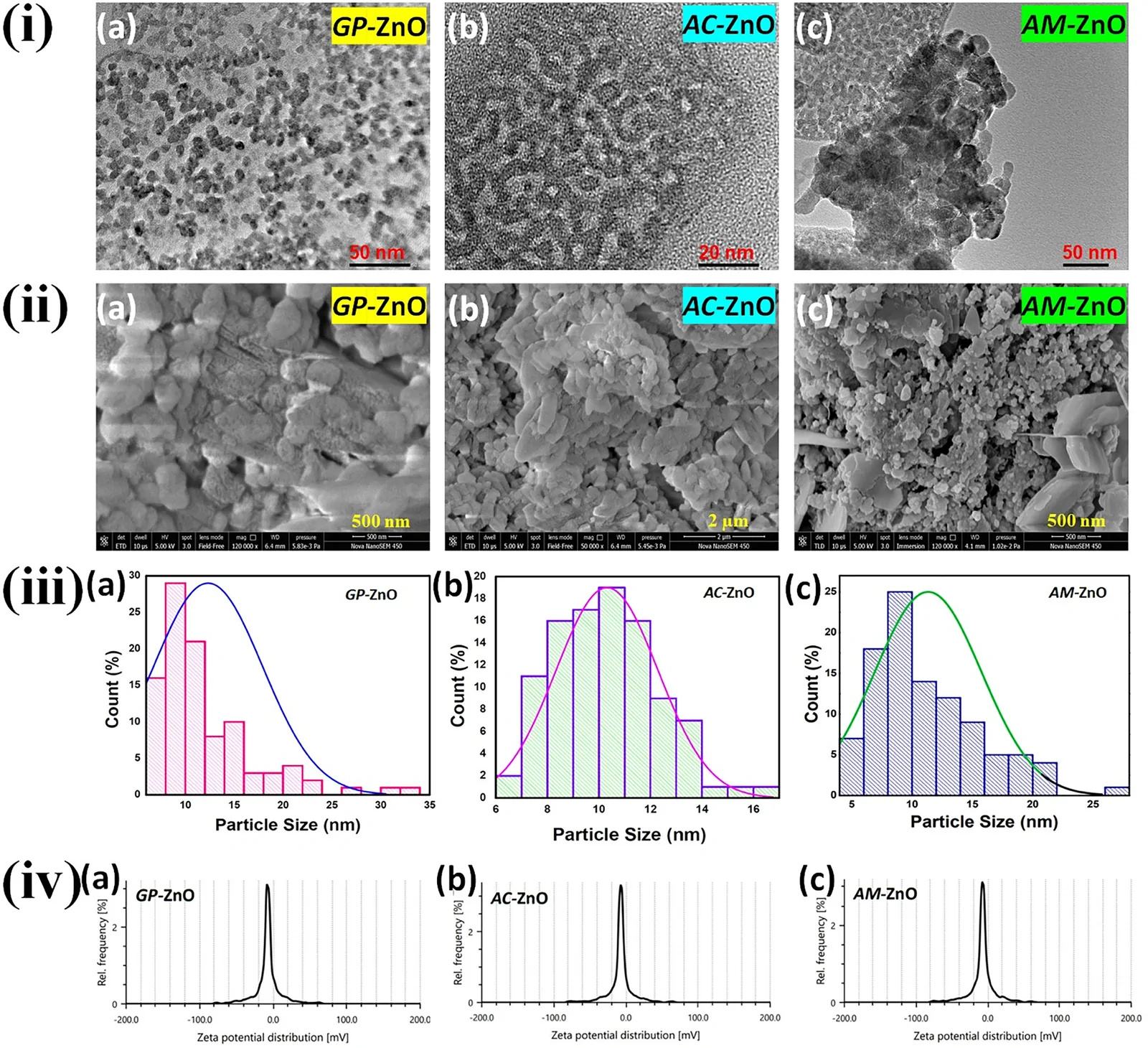
**(i**) High-resolution Transmission electron microscopy (HR-TEM) and **(ii)** Field-Emission scanning electron microscopy (FE-SEM) images of biosynthesized GP-ZnO, AM-ZnO, and AC-ZnO-NPs, showing predominantly spherical morphology and nanoscale dimensions. HR-TEM analysis indicated particle sizes ranging from 5 to 36 nm. **(iii)** Particle size distribution histograms of **(a)** GP-ZnO, **(b)** AC-ZnO, and **(c)** AM-ZnO nanoparticles, generated from HR-TEM image analysis using ImageJ software (NIH). n = 100 for GP-ZnO and AC-ZnO, and n = 96 for AM-ZnO. **(iv)** Zeta potential distributions of **(a)** GP-ZnO, **(b)** AC-ZnO, and **(c)** AM-ZnO NPs in aqueous dispersion.

We further performed zeta potential experiments to evaluate the surface charge attributes and colloidal stability of GP-ZnO, AC-ZnO, and AM-ZnO NPs in aqueous dispersion. The zeta potential distributions (**Figure 5iv**) showed a dominant, narrow peak centred in the negative potential range for all three formulations (GP-ZnO: −8.2 ± 0.3 mV; AC-ZnO: −8.6 ± 0.2 mV; and AM-ZnO: −8.6 ± 0.3 mV), with a minor secondary population extending toward more negative values (−50 to −75 mV). The preponderance of negative surface charge seems consistent with the presence of hydroxyl (-OH) and carboxyl (-COOH) functional moieties from residual plant materials adsorbed on the NPs surface, as well as additional hydroxylation of the ZnO-NPs. Zeta potential values ranging from −10 to −30 mV are generally associated with moderate colloidal stability (64, 65), however, the dominant zeta potential values observed for GP-ZnO, AC-ZnO, and AM-ZnO fall slightly outside this range, indicating limited electrostatic stabilization and a tendency toward some aggregation in aqueous dispersion as evident by electron microscopy (**Figure 5**).

### 3.6 Toxicity evaluation of ZnO-NPs

Evaluating toxicity against normal non-target cells is a pre-requisite for any novel formulation to ensure its safety profile. Keeping this in mind, the intrinsic toxicity profiles of various ZnO-NPs were established using healthy RBCs and a normal mammalian liver cell line before examining their anticancer properties. All plant extracts were found to be non-toxic to healthy RBC population. Upto 25 µg/mL, the ZnO-NPs demonstrated excellent hemocompatibility, however concentrations of 50 and 100 µg/mL resulted in a concentration-dependent increase in erythrocyte membrane damage. At the highest tested concentration of 100 μg/mL, haemolysis of 11.16±4.29, 12.03±5.58 and 15.78±4.87% was recorded in case of GP-ZnO, AM-ZnO, and AC-ZnO NPs, respectively (**Figure 6a**), compared with 66.28%±4.08% for doxorubicin. Further, all ZnO formulations demonstrated very little toxicity to normal mammalian liver lobe cells. Cell viability was reported to be 80.83±7.58, 81.36±6.68, 75.21±2.79% for GP-ZnO, AM-ZnO, and AC-ZnO NPs, respectively, at the highest tested concentration (100 μg/mL) (**Figure 6b**). Thus, it can be concluded that these ZnO-NPs did not exhibit any significant toxic manifestations against healthy, normal mammalian cells. Meanwhile, the known anticancer drug, Dox, exhibited significant toxicity to mammalian cell lines (35.75±6.92% viability).

**Figure 6:**
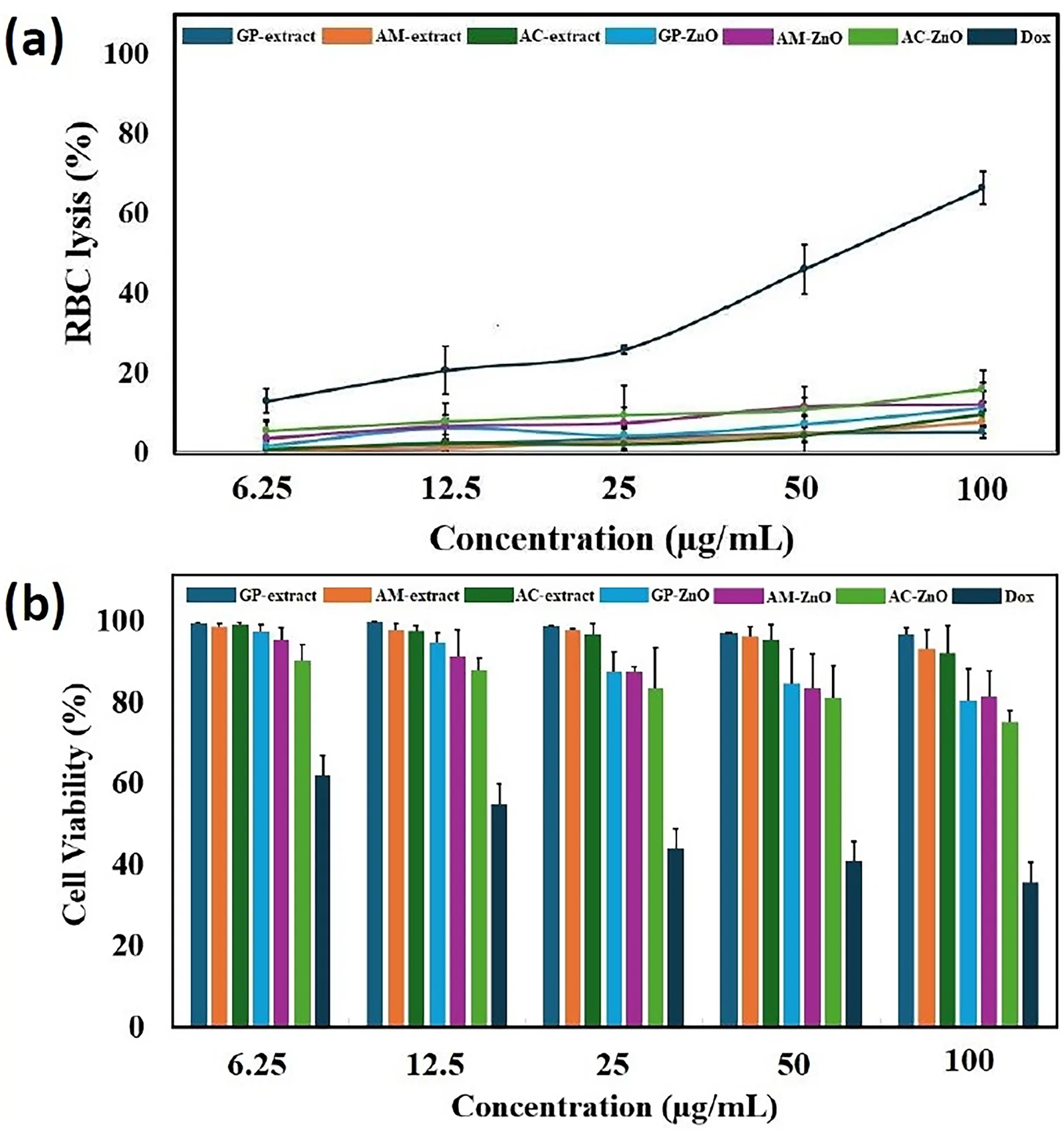
Toxicity studies of plant extracts and ZnO-NPs. **(a)** RBC lysis assay, **(b)** Effect for plant extracts and various ZnO nano-formulation of the viability of normal mammalian (THLE-2) cell lines. Doxorubicin served as a positive control.

One-way ANOVA in conjunction with Tukey’s HSD post-hoc analysis revealed a clear distinction between the groups and the Dox. No statistically significant differences were observed among GP-extract, AM-extract, AC-extract, GP-ZnO, AM-ZnO, and AC-ZnO (all adjusted *p ≥ 0.05*). In contrast, all treatments differed significantly from Dox, with GP-extract, AM-extract, AC-extract, GP-ZnO, AM-ZnO, and AC-ZnO showing highly significant differences compared with Dox (*p < 0.0001*). Among the ZnO formulations specifically, the differences between GP-ZnO and AM-ZnO, GP-ZnO and AC-ZnO, and AM-ZnO and AC-ZnO were not statistically significant, indicating comparable activity among the three biosynthesized ZnO-NPs formulations.

### 3.7 Anticancer activity of ZnO-NPs

MTT assay was performed to examine the anticancer attributes of the ZnO-NPs against HepG2 cells. It was observed that when treated with ZnO-NPs, metabolic activity of the cancer cells got reduced in a dose-dependent fashion (**Figure 7**), thereby reducing cell viability (66). For low concentrations (10 µg/mL), very little reduction in cell viability was observed (86-92%). However, a more prominent increase in cytotoxicity was observed for all formulations with the increment in the concentration to 50 µg/mL, viz. the cell viability was still 60.78±3.21, 58.95±4.09, and 55.95±3.82% for GP-ZnO, AM-ZnO, and AC-ZnO NPs, respectively. In case of the highest tested concentration (150 µg/mL), the cancer cell viability decreased to 23.05±2.17, 24.56±2.25 and 26.79±4.15%, indicating robust concentration-dependent cytotoxic action. Dox, a known anticancer agent, significantly inhibited the viability of HepG2 cell lines at all concentration tested. Numerically, although GP-ZnO showed the highest anticancer activity, the difference was not statistically significant compared with other ZnO-NPs. The significant reduction in cell viability underlines the therapeutic potential of these plant-derived ZnO-NPs as anticancer agents against liver cancer cells. However, the absence of plant extract-only and equimolar Zn^2+^ controls limits mechanistic interpretation; underscoring the need for further studies.

**Figure 7:**
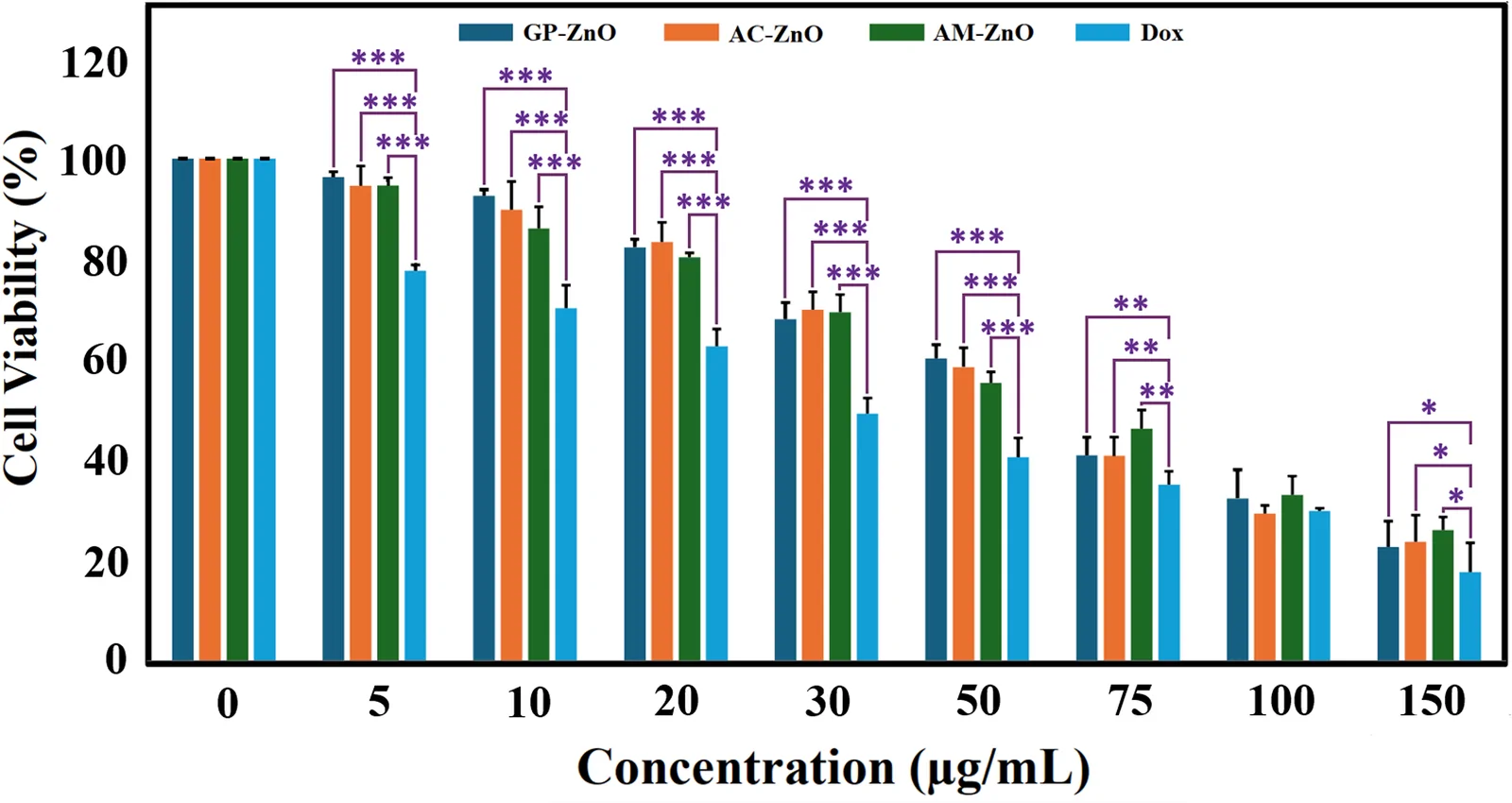
The dose-dependent cytotoxic effect of biosynthesized GP-ZnO, AM-ZnO, and AC-ZnO NPs on the HepG2 cancer cells as measured by the MTT test indicates that an increase in concentration of the NPs increases their cytotoxicity. p < 0.05, **p < 0.01, **p < 0.001 (one-way ANOVA with Tukey’s HSD post-hoc test), denoting statistically significant differences between the indicated groups at each concentration.

The IC_50_ values were calculated for each nano-formulation by fitting the experimental data to dose-response curves. The IC_50_ of GP-ZnO, AM-ZnO, and AC-ZnO NPs were found to be 60.25 ± 3.2, 68.35 ± 3.4, and 74.15 ± 2.6 µg/mL, respectively (**Table 3**). Dox displayed an IC_50_ value of 10.23 ± 1.35 µg/mL, similar to those reported earlier for HepG2 cells (67). These results indicate that all the tested NPs display only moderate cytotoxic activity against liver cancer cells. Variations observed in the cytotoxic response between different formulations can be explained due to different phytochemical profiles of the plant extracts. Since phytochemicals lose their integrity at 400°C, they are unlikely to directly affect the NPs’ cytotoxic action. However, phytochemicals may indirectly alter cytotoxicity by affecting NPs nucleation and production. It has been reported that the dislodging of the volatile phytoconstituents during calcination leaves defects or gaps in the ZnO crystal lattice (68). This formation of voids is greatly influenced by the chemical composition of the plant and can dictate the formation of ROS (67).

**Table 3:** The relative cytotoxic efficacy of the nano-phytotherapeutic formulations is shown by the half-maximal inhibitory concentration (IC_50_) of biosynthesized *GP-*ZnO*, AM-*ZnO, and *AC-*ZnO on HepG2 cells, after a 24-hour treatment.

S. No.	Sample	IC_50_ value (µg/mL)
1	Doxorubicin (DOX)	10.23 ± 1.35
2	GP-ZnO	60.25 ± 3.2
3	AM-ZnO	68.35 ± 3.4
4	AC-ZnO	74.15 ± 2.6

To rigorously assess the differences in cytotoxicity across the nano-formulations and Dox, ANOVA and Tukey’s HSD post-hoc test was executed. The calculated group means for cell viability (%) following treatment with the ZnO-NPs were closely clustered: GP-ZnO (X̄ = 66.39%), AM-ZnO (X̄ = 65.91%), and AC-ZnO (X̄ = 65.79%). Conversely, the Dox control group reached the lowest baseline cell viability mean value (X̄ = 53.95%). Pairwise comparisons among the nano-formulations yielded negligible differences in cytotoxicity and uniform, capped adjusted probability values (GP-ZnO vs. AC-ZnO; GP-ZnO vs. AM-ZnO; AC-ZnO vs. AM-ZnO: all *p=1.00*). These data demonstrate that varying the botanical extract during the green synthesis framework introduces no statistically distinct variation in final biological performance or cell survival dynamics. A noticeable raw downward shift in cell viability was observed when comparing the NPs treatments against the standard Dox control (ΔX̄ ≈ 12%). However, under the strict family-wise error rate protections of Tukey’s multiple comparison test, this variance failed to attain substantial significance at the conventional α = 0.05 threshold.

The anticancer effects observed in the current investigation seems consistent with other researchers who have revealed the therapeutic potential of ZnO-NPs in various cancer types. Suggestively, Zhang et al. have detected dose-dependent variations cytotoxic reactions in T24 bladder cancer cells exposed to ZnO-NPs, even at very low concentrations of the particles (66). On the same note, Karimzadeh and coworkers utilized *Acantholimon serotinum* extract to produce ZnO-NPs that selectively exerted cytotoxic effects on the majority of cancer cell lines but exhibited relatively moderate toxicity to normal cells (69). Further, Berehu et al. demonstrated that ZnO-NPs synthesized using *Swertia chirayita* exhibited significant inhibitory effects on tumor cell lines such as HCT-116 and Caco-2 (70).

Numerous studies have established that ZnO-NPs exhibit excellent biocompatibility and a favourable safety profile at therapeutically relevant doses, owing to the biodegradability of ZnO into physiologically regulated Zn^2+^ ions. While bulk ZnO is recognized by the FDA as Generally Recognized As Safe (GRAS) (71), recent *in-vitro* studies have consistently reported minimal cytotoxicity toward normal mammalian cells, including fibroblasts, keratinocytes, endothelial cells, and HEK-293 cells, with high cell viability at concentrations that selectively induce cancer cell death (72–74). In addition, haematology evaluations have demonstrated minimal hemolysis while maintained RBC membrane integrity, especially for green-synthesized ZnO-NPs (75). Similar studies have shown no significant alteration in white blood cells count, suggesting negligible immunotoxicity (76). As expected, Dox causes a concentration-dependent RBC lysis, demonstrating its intrinsic haemolytic property (77). Furthermore, evidences of genotoxicity, carcinogenic effects, or reproductive toxicity of ZnO-based nanoformulations in humans has been lacking (78, 79).

### 3.8 Reactive oxygen species studies

To understand the mechanism behind the dose-dependent cytotoxic potential of ZnO-NPs against HepG2 cancer cells, intracellular ROS accumulation was examined using DCFH-DA dye. As seen in **Figure 8i**, a heightened fluorescence response was observed in all NPs-treated groups when compared with untreated controls, indicating substantial intracellular ROS accumulation after cancerous cells were exposed to ZnO-NPs. This elevated fluorescence emission was associated with typical apoptotic morphological alterations, including cell shrinkage, membrane disintegration, and diminished cell density, while control cells exhibited little fluorescence and maintained typical cellular morphology, indicative of baseline ROS levels.

**Figure 8:**
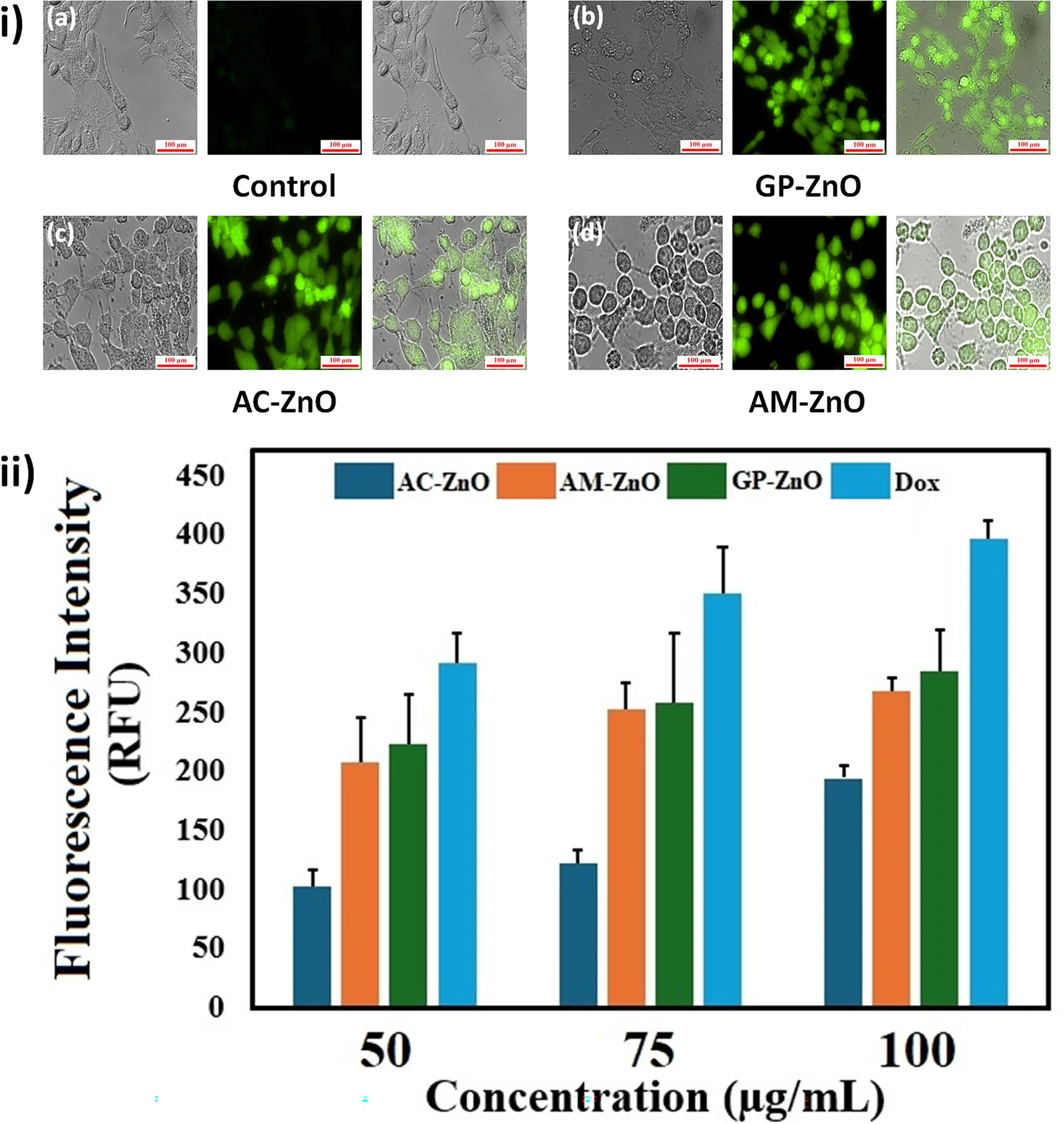
**(i)** Fluorescence confocal microscopy images of intracellular ROS level of cells, including HepG2: **(a)** the untreated control; **(b)** the GP-ZnO-treated; **(c)** the AC-ZnO-treated; and **(d)** the AM-ZnO-treated. As biosynthesized ZnO-NPs generate green fluorescence, the intensity level of the fluorescence is greater, which means that the level of ROS and oxidative stress is much higher. Each scale bar corresponds to 100 μm **(ii)** Dose-dependent intracellular reactive oxygen species (ROS) generation in HepG2 cells following treatment with GP-ZnO, AM-ZnO, AC-ZnO nanoparticles, and doxorubicin (DOX).

Quantitative fluorescence analysis further confirmed these observations. The positive control (Dox) exhibited the highest fluorescence intensities across all tested concentration range, validating the sensitivity of the assay, while the negligible fluorescence observed in the blank medium confirmed that the measured signal originated from intracellular ROS production. The RFU values for GP-ZnO, AM-ZnO and AC-ZnO at a concentration of 50 μg/mL was found to be 223.0, 207.6 and 102.7 RFU, respectively. At 75 μg/mL, GP-ZnO produced the highest ROS levels (258.86 RFU), closely followed by AM-ZnO (252.81 RFU), while AC-ZnO (122.25 RFU) showed lowest RFU among ZnO-NPs as shown in **Figure 8ii**. Furthermore, at 100 µg/mL, intracellular ROS levels increased to 285.63, 268.27, and 194.60 RFU for GP-ZnO, AM-ZnO, and AC-ZnO, respectively. The elevated ROS generation suggests that oxidative stress may contribute to the anti-cancer effects of ZnO-NPs. Excessive ROS disrupts cellular redox homeostasis, causing lipid, protein, and DNA damage, mitochondrial dysfunction, and activation of apoptotic signalling pathways, ultimately leading to programmed cell death in HepG2 cells (80, 81).

One-way ANOVA demonstrated a significant overall difference among treatments. Tukey’s post-hoc analysis revealed that both GP-ZnO (*p = 0.0399*) as well as DOX (*p = 0.0015*) generated significantly higher ROS levels than AC-ZnO. However, the differences between GP-ZnO and AM-ZnO (*p = 0.9808*), GP-ZnO and DOX (*p = 0.1114*), and AM-ZnO and DOX (*p = 0.0656*) were not statistically significant. Although GP-ZnO showed the highest numerical ROS generation among the three ZnO formulations, its overall ROS response was statistically comparable to that of AM-ZnO and DOX.

### 3.9 Annexin V apoptosis assay

The results from the Annexin V-FITC flow cytometric assay demonstrate that the biosynthesized ZnO-NPs induce an increase in Annexin V^+^ cells (**Figure 9**), indicating enhanced PS externalization, a hallmark early marker of apoptotic cell death (82). The unstained control (0.00%) was used to establish the fluorescence-negative region and assess background autofluorescence. Untreated cells exhibited a low Annexin V^+^ population (1.20%), indicating minimal PS externalization under basal conditions. In contrast, all NPs-treated groups exhibited a significant increase of Annexin V^+^ cells, representing the externalization of PS. The highest apoptotic populations were obtained for GP-ZnO (59.71%) and AM-ZnO (57.40%) among the tested formulations, which were even higher than the apoptosis induced by Dox (50.05%), with AC-ZnO also showing considerable ability to cause apoptosis at 36.91%. Taken together with cytotoxicity and ROS data, these findings support the involvement of apoptotic processes in ZnO-NP-induced HepG2 cell death, with ROS accumulation and oxidative stress potentially contributing to these effects. However, ROS generation does not appear to be the sole determinant of ZnO-NP-induced cellular toxicity. Further, because a viability discriminator such as PI or 7-AAD was not included, late apoptotic cells could not be distinguished from necrotic cells, and therefore the precise mode of cell death requires further investigation.

**Figure 9:**
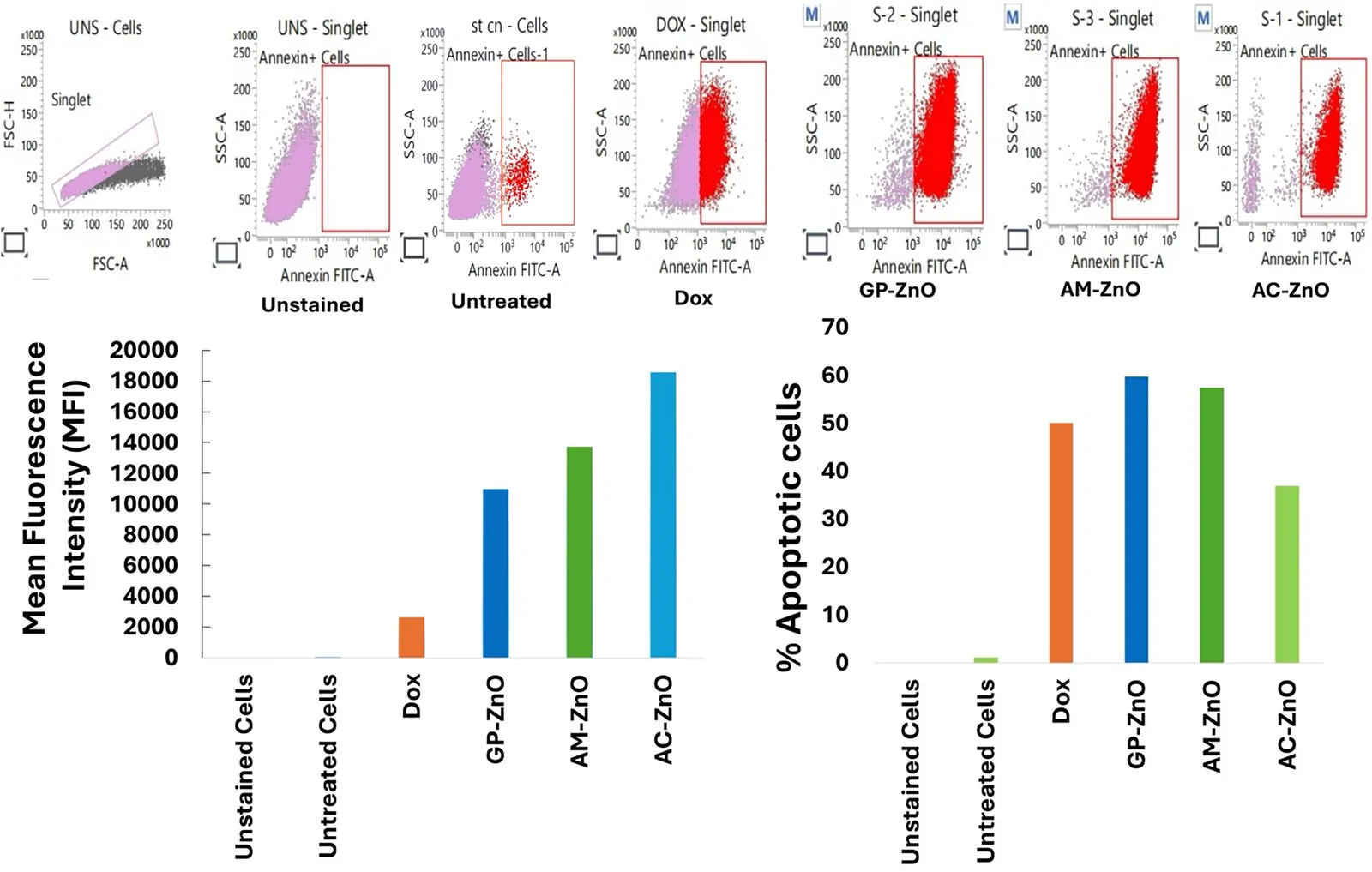
Annexin V-FITC flow cytometric analysis of apoptosis in HepG2 cells treated with ZnO-NPs. Representative flow cytometry plots and corresponding bar graphs show mean fluorescence intensity (MFI) and apoptotic cell percentages in unstained, untreated, doxorubicin-treated, and GP-ZnO-, AM-ZnO-, and AC-ZnO-treated cells. ZnO-NPs significantly increased Annexin V fluorescence and apoptosis compared with untreated cells.

The present findings resonated with numerous reports demonstrating that ZnO-NPs induce apoptosis in HC through ROS-dependent activation of the intrinsic mitochondrial pathway. Synergistic effects have previously been reported in the case of biosynthesized ZnO-NPs from the extracts of *Aquilegia pubiflora* (83), *Salvadora persica* (84), and coffee leaves (85) wherein significantly high positivity for annexin-V, ROS generation, DNA fragmentation, and mitochondrial depolarization was observed (86). Elevated intracellular ROS promotes oxidative damage to mitochondrial membranes, leading to dissipation of mitochondrial membrane potential, cytochrome *c* release, activation of caspase-9 and caspase-3, and ultimately phosphatidylserine externalization, which is detected by Annexin V staining (87–90). Li et al. further demonstrated that ZnO-NPs significantly increased Annexin V^+^ HepG2 cells while simultaneously upregulating the pro-apoptotic proteins Bax and p53, suppressing Bcl-2 expression, and activating the caspase cascade, thereby confirming mitochondrial apoptosis as the dominant mechanism of ZnO-NPs-mediated cytotoxicity (91). Similar observations were reported where nanosized ZnO selectively induces oxidative stress and apoptosis in cancer cells through mitochondrial dysfunction rather than nonspecific membrane lysis (90, 92). Further, it has been proven that ZnO-NPs trigger ROS-mediated apoptosis in human cancer cells via activation of p53 and caspase-dependent signalling pathways while exhibiting relatively lower toxicity toward normal cells (93, 94).

An intriguing observation of this study was that the differential ROS and Annexin V responses indicate that ROS generation alone does not fully account for the observed cytotoxic effects. Although all three ZnO-NPs generated lower intracellular ROS levels than Dox, GP-ZnO and AC-ZnO induced higher Annexin V^+^ cells, with AC-ZnO showing relatively low ROS despite the second-highest Annexin V^+^ count. This indicates that ROS is not the sole driver of ZnO-NPs-mediated apoptosis. Following cellular internalization, ZnO-NPs release Zn^2+^ ions, which disrupt mitochondrial function, activate caspase-dependent apoptotic pathways, and induce endoplasmic reticulum stress and calcium imbalance, thereby promoting apoptosis through both ROS-dependent and ROS-independent mechanisms (95, 96). Moreover, localized mitochondrial ROS, which may not be fully captured by DCFH-DA assays (97), can efficiently initiate apoptotic signalling. Thus, the enhanced apoptosis induced by ZnO-NPs likely results from the coordinated activation of multiple cell death pathways rather than high ROS production alone.

## 4 Conclusion

Herein, we report the green synthesis of ZnO-NPs by using aqueous extracts of *G. parviflora*, *A. conyzoides* and *A. mexicana*. These neglected plants are proposed in this study as sustainable resources for nanomedicine. The phytochemicals present in the extracts acted as natural reducing and stabilizing agents, resulting in phase-pure, crystalline, almost spherical, nano-sized ZnO-NPs with a hexagonal wurtzite structure. Among the tested ZnO-NPs, GP-ZnO showed the highest numerical cytotoxicity, although differences among the NPs were not statistically significant. Further, the observed increases in ROS and Annexin V^+^ cells suggest the involvement of oxidative stress and apoptotic processes. Notably, the apoptotic response did not consistently parallel ROS generation, suggesting that ROS generation alone may not fully explain the observed cytotoxic effects. Additionally, Annexin V staining without PI or 7-AAD cannot distinguish a contribution from late apoptosis and/or necrotic cell death, warranting further mechanistic validation. Furthermore, the lack of plant extract controls and equimolar Zn^2+^ controls makes it difficult to distinguish the individual contributions of residual phytoconstituents and released Zn^2+^ to the observed effects. Future studies incorporating these controls, along with *in-vivo*, pharmacokinetic, and safety assessments, are warranted to clarify the underlying mechanisms and evaluate the translational potential of plant-mediated ZnO-NPs as eco-friendly anticancer nanotherapeutics.

## Data Availability

The raw data supporting the conclusions of this article will be made available by the authors, without undue reservation.
